# Improved Etiological Diagnosis of Nonresolving or Slowly Resolving Pneumonia Through Combined Endobronchial Ultrasound-Guided Biopsy and Metagenomic Sequencing

**DOI:** 10.1155/carj/7651699

**Published:** 2025-08-28

**Authors:** Qiang Li, Li Jian, Qiquan Zhao

**Affiliations:** ^1^Department of Respiratory and Critical Care Medicine, The Affiliated Dazu's Hospital of Chongqing Medical University, Chongqing, China; ^2^Department of Endocrinology, The Affiliated Dazu's Hospital of Chongqing Medical University, Chongqing, China

**Keywords:** diagnostic yield, endobronchial ultrasound, metagenomic next-generation sequencing, nonresolving or slowly resolving pneumonia, transbronchial lung biopsy, tuberculosis

## Abstract

**Background:** Nonresolving or slowly resolving pneumonia (NRP) poses a diagnostic challenge because infectious and noninfectious etiologies often mimic community-acquired pneumonia on imaging. Endobronchial ultrasound-guided transbronchial lung biopsy (EBUS-TBLB) improves tissue acquisition for peripheral lesions, whereas metagenomic next-generation sequencing (mNGS) offers culture-independent pathogen detection. Whether their combination enhances etiological clarification of NRP remains uncertain.

**Methods:** A total of 109 consecutive adults with NRP unresponsive to standard antimicrobial therapy were randomized to EBUS-TBLB alone (*n* = 66) or EBUS-TBLB + mNGS (*n* = 43). Baseline characteristics, diagnostic yield, and procedure-related complications were recorded. Diagnostic positivity, sensitivity for infectious agents, and safety profiles were compared using *χ*^2^ or Fisher's exact tests, with *p* < 0.05 considered significant.

**Results:** Overall diagnostic yield increased from 50.0% with EBUS-TBLB to 72.1% with the combined approach (*χ*^2^ = 4.37, *p* < 0.05). mNGS significantly improved detection of bacterial/fungal pneumonia (0% vs. 13.9%; *p* < 0.05) and pulmonary *tuberculosis* (0% vs. 20.9%; *p* < 0.05). Malignancy remained the predominant diagnosis (57.8% of all cases); yields for most tumor subtypes were comparable between groups. Complication rates did not differ between the two groups: minor bleeding (19.7% vs. 23.3%), hypoxia (50.0% vs. 48.8%), pneumothorax (4.5% vs. 0%), and delayed recovery (4.5% vs. 7.0%) (*p* > 0.05). No severe adverse events occurred.

**Conclusions:** EBUS-TBLB + mNGS represents a paradigm shift in the diagnosis of complex respiratory cases, integrating imaging with advanced genomics to enhance precision medicine. In practice, early implementation of the EBUS-TBLB + mNGS diagnostic protocol in patients with NRP can help exclude malignancy or confirm an infectious etiology.

## 1. Introduction

**Figure 1:**
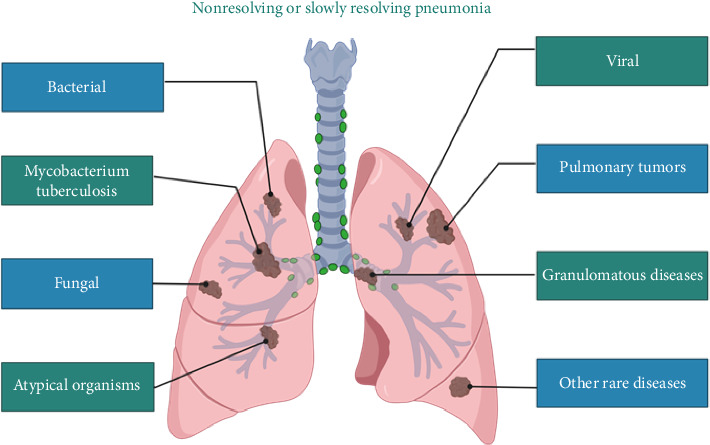
The causes of NRP include bacterial infections, *tuberculosis*, fungal infections, viral infections, atypical pathogens, pulmonary tumors, granulomatous diseases, and other rare diseases.

In 1975, the concept of “nonresolving or slowly resolving pneumonia (NRP)” was first proposed, defined as a pneumonia-like illness persisting for more than 21 days [1]. In current clinical practice, this term typically refers to pneumonia-like manifestations that persist or even progress on imaging despite standard antibiotic therapy. Such manifestations include many noninfectious diseases whose clinical and radiological features closely resemble those of community-acquired pneumonia (CAP)—for example, malignant tumors such as invasive adenocarcinoma, pulmonary lymphoma, and leukemia, as well as benign conditions such as pulmonary hemorrhage, eosinophilic pneumonia, pulmonary edema, and organizing pneumonia. The incidence of this kind of protracted, nonresolving pneumonia is not negligible. According to reports, approximately 10% of CAP cases have a poor treatment response, and in hospital-acquired pneumonia (HAP), this proportion exceeds 30%; furthermore, pneumonia that does not respond to treatment has a mortality rate three times higher in CAP and five times higher in HAP [2]. It is estimated that 8% of bronchoscopic examinations and 15% of inpatient respiratory consultations are related to pneumonia that remains unresolved after treatment [3]. In the past, clinicians might opt for close observation in patients whose pneumonia failed to resolve after initial therapy. However, failing to promptly identify the underlying cause can lead to serious consequences. If the cause is infectious, the disease course may become prolonged and refractory, with recurrent exacerbations, residual structural damage or functional impairment, or even progression to septic shock and death. If the cause is noninfectious, it can result in a protracted course, secondary infections, or dissemination of tumor cells, thereby missing the optimal window for treatment. The reasons for pneumonia persisting without resolution are complex and varied. It may be due to infection by drug-resistant or uncommon pathogens (such as atypical bacteria, *tuberculosis*, fungi, viruses, or other uncommon microorganisms), or it may be a noninfectious condition misdiagnosed as pneumonia (such as pulmonary tumors, granulomatous diseases, and other rare diseases) (Figure 1). Currently, no large-scale epidemiological data on NRP are available domestically or internationally. However, combining findings from the existing studies and pneumonia management guidelines, the most common causes of nonresolving pneumonia are lung cancer and pulmonary *tuberculosis* [4–6]. In recent years, endobronchial ultrasound-guided transbronchial lung biopsy (EBUS-TBLB) has been widely used for the diagnosis of pulmonary lesions and has demonstrated a significant clinical value. Previous studies show that EBUS is superior to conventional bronchoscopy, significantly improving the diagnostic yield for peripheral lung lesions. Metagenomic next-generation sequencing (mNGS) is a technology that utilizes high-throughput sequencing to rapidly analyze all nucleic acids in a sample and compare them to genomic databases of various organisms, thereby identifying the types and proportions of microorganisms present. This technique can detect all DNA or RNA in a clinical specimen and simultaneously identify bacteria, viruses, fungi, and parasites, demonstrating important clinical utility [7, 8]. Theoretically, combining EBUS-TBLB with mNGS testing could significantly improve the diagnostic yield for both infectious and noninfectious causes of pneumonia that resolve poorly and thus become an important diagnostic tool for clinicians. Unfortunately, there are currently no studies explicitly evaluating the clinical value of EBUS-TBLB combined with mNGS for diagnosing NRP. The aim of this study was to investigate the distribution of etiologies and determine the clinical utility of EBUS-TBLB + mNGS in the early diagnosis of NRP. This study introduces a novel multimodal diagnostic strategy that combines EBUS-TBLB with mNGS. To our knowledge, it is the first large-scale study, nationally or internationally, to systematically evaluate the clinical value of this combined approach. Traditional diagnostic methods often fall short when confronted with unexplained pulmonary lesions; by integrating interventional imaging with advanced molecular diagnostics, our approach significantly enhances pathogen detection. This innovative cross-disciplinary integration not only exemplifies the application of precision medicine in respiratory care but also fills a critical gap in the existing literature.

## 2. Materials and Methods

### 2.1. Clinical Data

An analysis was performed on 131 patients admitted to our hospital between January 2021 and September 2024. All patients had an initial diagnosis of pneumonia and showed persistent pulmonary lesions despite routine and intensive anti-infective therapy. They were randomly allocated to two diagnostic groups based on the technique used: EBUS-TBLB alone vs. EBUS-TBLB + mNGS. Patient characteristics, diagnostic outcomes, and specific procedural parameters were recorded. The diagnostic positivity rates, sensitivity, and complication rates were compared between the two groups. All patients provided informed consent, and any patient with uncontrolled blood pressure was medically managed before bronchoscopy. *Inclusion criteria*: NRP was defined as (1) persistent clinical symptoms (fever, cough, or dyspnea) for ≥ 21 days despite appropriate antibiotic treatment and (2) ≤ 50% reduction in pulmonary lesion size or evidence of persistent/progressive infiltrates on chest imaging within the same timeframe. *Exclusion criteria*: Patients were excluded if they (1) could not tolerate routine bronchoscopy, (2) exhibited poor cooperation or had a diagnosed psychiatric disorder, (3) had severe cardiopulmonary dysfunction, (4) had active bleeding, (5) showed abnormal platelet function or coagulation disorders, or (6) had incomplete clinical data. The study protocol was approved by the Institutional Ethics Committee.

### 2.2. Methods

#### 2.2.1. Chest Imaging Interpretation

Chest HRCT or contrast-enhanced CT scans of patients with pneumonia unresponsive to treatment were independently interpreted by a senior respiratory physician and a radiologist.

#### 2.2.2. Apparatus

The endobronchial ultrasound equipment used was a radial probe EBUS (RP-EBUS) system, along with disposable biopsy needles manufactured by Olympus (Japan).

#### 2.2.3. Procedure

Preoperative preparation was carried out according to the standard requirements for bronchoscopy, and all procedures strictly adhered to the guidelines for the Application of Diagnostic Flexible Bronchoscopy. RP-EBUS was performed using a 20-MHz radial probe (Olympus BF-UC260FW) inserted through a 2.0-mm working channel bronchoscope. Biopsies were obtained via 1.9-mm disposable forceps (Olympus FB-221D) under real-time ultrasound guidance. For patients in the EBUS-TBLB group, biopsy tissue samples were sent for pathological and cytological examination, routine bacterial and fungal cultures, acid-fast *bacillus* staining, and *Mycobacterium tuberculosis* nucleic acid testing. For patients in the EBUS-TBLB + mNGS group, in addition to all of the above tests, a portion of each biopsy sample was also sent for mNGS analysis.

#### 2.2.4. Clinical Diagnostic Criteria

Malignant tumors: It was confirmed by the presence of tumor cells on cytology or by histopathological evidence of malignancy. Infectious diseases: It was established by clear etiological evidence from tests such as mNGS, routine bacterial/fungal cultures, acid-fast *bacillus* staining, or *Mycobacterium tuberculosis* nucleic acid testing, combined with pathology to exclude neoplasm. Tuberculosis: It was confirmed by pathological evidence of caseating epithelioid granulomas or a positive result for acid-fast bacilli or *Mycobacterium tuberculosis* DNA. Other benign diseases: They are mainly reactive lymphoid hyperplasia or chronic inflammation. Other diseases must be excluded and a follow-up chest CT at 6 months shows the lesion has decreased or remained unchanged in size to confirm the diagnosis.

### 2.3. Statistical Methods

The statistical analysis was performed using SPSS 29.0 software. Non-normally distributed measurement data were described using medians and were analyzed via the rank-sum test. Normally distributed measurement data were expressed as the mean ± SD. Two-sample comparisons were performed using the *t*-test, and comparisons among multiple samples were analyzed using ANOVA; categorical data were analyzed via the *χ*^2^ test. A *p* value of < 0.05 was considered statistically significant.

## 3. Results

### 3.1. Patient Enrollment and Attrition

**Figure 2:**
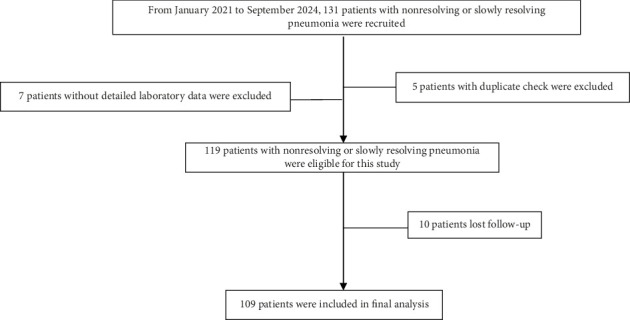
Study flowchart.

Between January 2021 and September 2024, 131 consecutive patients with NRP were screened for eligibility (Figure 2). Seven patients were excluded due to incomplete laboratory data, and five were removed due to duplicate records, leaving 119 eligible participants. During follow-up, ten patients were lost, resulting in 109 individuals (83.2% of those initially screened) in the final analysis.

### 3.2. Baseline Clinical Data

A total of 109 patients were included in the study, with 66 in the EBUS-TBLB group and 43 in the EBUS-TBLB + mNGS group. Their baseline characteristics are presented in Table 1. There were no statistically significant differences between the two groups in sex, age, or lesion size (*p* > 0.05).

### 3.3. Diagnostic Results and Rates With Various Parameters

Among the 109 patients analyzed, malignant tumors predominated (57.8%), whereas infectious lesions accounted for 14.7% (9.2% bacterial/fungal pneumonia and 5.5% pulmonary *tuberculosis*). Overall diagnostic yield increased from 50.0% with conventional EBUS-TBLB (33/66) to 72.1% when mNGS was added (31/43; *p* < 0.05). The combined approach markedly improved the detection of bacterial/fungal pneumonia and *tuberculosis* (*p* < 0.01) without compromising the identification of most malignant subtypes. However, the numerically higher yield of adenocarcinoma in the EBUS-TBLB group likely reflects the limited sample size rather than a true advantage. Detection rates for lung squamous cell carcinoma, small cell lung cancer, and other rare tumors were comparable between the two groups. These findings demonstrate that integrating mNGS into EBUS-guided biopsy substantially enhances overall diagnostic clarity, chiefly by revealing otherwise occult infectious etiologies (Table 2).

### 3.4. Incidence of Complications

Among the 109 patients who underwent bronchoscopy with either local anesthesia or a laryngeal mask under general anesthesia, in the EBUS-TBLB group, minor bleeding occurred in 13 patients (19.7%), hypoxia in 33 patients (50.0%), pneumothorax in 3 patients (4.5%), and delayed recovery in 3 patients (4.5%). In the EBUS-TBLB + mNGS group, minor bleeding was observed in 10 patients (23.3%), hypoxia in 21 patients (48.8%), pneumothorax in 0 patients (0%), and delayed recovery in 3 patients (7.0%). No severe complications such as mediastinal emphysema, major vascular injury, shock, or death were observed in either group. There were no significant differences in complication rates between the two groups (*p* > 0.05) (Table 3).

## 4. Discussion

EBUS is a bronchoscopic technique that uses ultrasound to visualize the airway wall, lungs, and mediastinal structures. It is known for its convenience, effectiveness, minimal invasiveness, and safety [9, 10]. In the 1990s, EBUS was first applied to bronchial diseases. By introducing a miniature ultrasound probe through the bronchoscope to obtain real-time imaging, EBUS can effectively distinguish lesions from surrounding tissues and blood vessels, significantly improving the diagnostic yield for peripheral lung lesions while reducing surgical risk. Currently, EBUS is categorized into two types: convex probe EBUS (CP-EBUS) and radial probe EBUS (RP-EBUS). CP-EBUS provides an ultrasound image in a plane parallel to the bronchoscope's long axis and can be used to perform needle biopsies of mediastinal lymph nodes—a procedure commonly known as EBUS–transbronchial needle aspiration (TBNA). RP-EBUS, in contrast, provides detailed, high-resolution 360° images of the airway wall and surrounding structures. It is used to visualize peripheral pulmonary lesions and to guide bronchoscopists in performing transbronchial lung biopsies, a technique clinically referred to as EBUS-TBLB. Studies have shown that EBUS-TBLB significantly enhances diagnostic sensitivity and has a substantial diagnostic value for peripheral lung lesions [11, 12]. In clinical practice, however, the etiology of an unresolved pneumonia is often unclear. Although endobronchial ultrasound can diagnose most malignant tumors and benign granulomatous diseases, the causes of many infections remain undetermined, leading to suboptimal treatment outcomes or misdiagnosis as neoplastic disease. EBUS offers unparalleled advantages in specimen acquisition, enabling real-time visualization of lung lesions and the collection of tissue samples for accurate diagnosis. Additionally, mNGS enables comprehensive detection of all DNA and/or RNA present in clinical samples, thereby facilitating the identification of infectious pathogens [13, 14]. Compared with conventional DNA-based diagnostic tools such as multiplex PCR or 16S rRNA sequencing, mNGS offers a hypothesis-free and unbiased approach to detect a broad spectrum of pathogens, including bacteria, fungi, viruses, and atypical organisms in a single assay. Its high sensitivity and comprehensive coverage significantly improve diagnostic yield, especially in polymicrobial infections and rare pathogens. Previous studies have reported that combining EBUS-TBLB with mNGS yields an overall diagnostic positivity of up to 90.83% for NRP, with malignancies (57.8%) and infectious diseases (20.18%) being the predominant causes; notably, *tuberculosis* accounts for 7.34% of the infections. By comparison, when EBUS-TBLB is used alone for pulmonary nodules, its diagnostic yield is around 80.4%. Incorporating molecular tests such as Xpert MTB/RIF and NGS can further improve the sensitivity and specificity of *tuberculosis* detection (area under the curve = 0.826) [15].

NRP is a common clinical entity encountered in practice. Notably, the complication rates of pneumothorax (0%–8%) and bleeding (0.8%) associated with EBUS-TBLB are much lower than those observed with CT-guided percutaneous lung biopsy (21.4% pneumothorax and 28.5% hemoptysis) [16, 17]. The results of our study indicate that EBUS-TBLB + mNGS is a highly safe and effective diagnostic approach. The main complications observed were minor bleeding, hypoxia, pneumothorax, and delayed recovery, with no severe complications such as mediastinal emphysema, major vascular injury, shock, or death. This underscores the procedure's safety and efficacy as a diagnostic tool. Based on the characteristics of the two types of EBUS, we suggest that the combined use of EBUS-TBLB and mNGS be considered especially for patients with predominantly peripheral lung lesions, in order to increase diagnostic accuracy and reduce complications. Our findings also suggest that EBUS-TBLB + mNGS should be employed early in the course of NRP to exclude neoplastic disease or identify an infectious etiology, particularly when traditional culture methods fail to identify the pathogen.

This study has several inherent limitations, including the fact that we did not perform a predetermined sample size calculation, which may introduce selection bias and limit the generalizability of our findings. Nevertheless, we enrolled all eligible patients (*n* = 109) during the study period, making our cohort one of the largest reported for “refractory” pneumonia. The lack of a prespecified sample size is justified by the exploratory nature of our analysis, which was designed to capture real-world clinical experience rather than test a specific hypothesis. Importantly, our relatively large sample size enabled us to detect significant differences in key outcomes (such as diagnostic yield), thereby reducing the risk of a Type II error. Although the single-center design is a limitation, nonresolving pneumonia is a relatively uncommon condition, and our center—a specialized respiratory facility with a high volume of complex cases—provided a representative sample for similar clinical settings. Previous studies using EBUS combined with mNGS for diagnosing pulmonary infections have typically involved only a few dozen cases; in contrast, our cohort of over one hundred cases provides robust statistical power to support our conclusions. To mitigate retrospective bias, we employed continuous enrollment of all eligible patients and ensured data accuracy through independent double data extraction. Future investigations should adopt multicenter, prospective designs with larger sample sizes to validate and extend these findings.

## 5. Conclusions

In conclusion, EBUS-TBLB + mNGS is an effective method for diagnosing the etiology of NRP. It offers excellent safety, significantly reduces the time required for lesion localization and the procedure, and improves diagnostic accuracy and sensitivity. This combined approach has the potential to improve patient outcomes and represents a novel diagnostic modality in respiratory disease in recent years. It is an effective advance in diagnostic capability for respiratory infections and tumors and merits broader clinical adoption. In practice, early implementation of the EBUS-TBLB + mNGS diagnostic protocol in patients with NRP can help exclude malignancy or confirm an infectious etiology, especially when traditional culture methods fail to identify a pathogen.

## Figures and Tables

**Table 1:** Baseline characteristics of study population.

Item	EBUS-TBLB (*N* = 66)	EBUS-TBLB + mNGS (*N* = 43)	*t*/*χ*^2^	*p*
Gender (male/female, *N*)	43/23	31/12	*χ* ^2^ = 0.30	0.58
Age (year, *χ* ± *s*)	67.14 ± 8.02	63.63 ± 11.81	*t* = 1.71	0.09
Lesion size (mm, *χ* ± *s*)	32.21 ± 5.62	32.33 ± 6.09	*t* = 0.10	0.92

**Table 2:** Diagnostic results and rates with various parameters.

Item	EBUS-TBLB (*N* = 66)	EBUS-TBLB + mNGS (*N* = 43)	Test	*χ* ^2^/*F*	*p*
Total number of diagnosis (%)	33 (50.00)	31 (72.09)	*χ* ^2^	4.37	0.037^∗^
Bacterial or fungal pneumonia (%)	0 (0)	6 (13.95)	Fisher	—	0.003^∗^
Pulmonary *tuberculosis* (%)	0 (0)	9 (20.93)	Fisher	—	0.00013^∗^
Lung adenocarcinoma (%)	27 (40.91)	7 (16.28)	*χ* ^2^	6.26	0.012^∗^
Lung squamous cell carcinoma (%)	3 (4.55)	3 (6.98)	Fisher	—	0.679
Small-cell lung cancer (%)	1 (1.52)	3 (6.98)	Fisher	—	0.298
Other malignant tumors (%)	2 (3.03)	3 (6.98)	Fisher	—	0.381

*Note:χ* ^2^ tests were continuity-corrected when appropriate; Fisher's exact test was two-tailed.

^∗^Statistically significant differences (*p* < 0.05).

**Table 3:** Incidence of complications.

Item	EBUS-TBLB (*N* = 66)	EBUS-TBLB + mNGS (*N* = 43)	Test	*χ* ^2^/*F*	*p*
Minor bleeding (%)	13 (19.69)	10 (23.26)	*χ* ^2^	0.042	0.838
Hypoxia (%)	33 (50.00)	21 (48.84)	*χ* ^2^	< 0.001	1
Pneumothorax (%)	3 (4.55)	0 (0)	Fisher	—	0.277
Delayed recovery (%)	3 (4.5)	3 (6.98)	Fisher	—	0.679

*Note:χ* ^2^: Yates' continuity-corrected Chi-square test; Fisher: two-tailed Fisher's exact test.

## Data Availability

All data are fully available without restriction.

## References

[1] Kuru T., Lynch J. P. (1999). Nonresolving or Slowly Resolving Pneumonia. *Clinics in Chest Medicine*.

[2] Sun H., Chen R., Li T. (2023). Combination of Transbronchial Cryobiopsy Based Clinic-Radiologic-Pathologic Strategy and Metagenomic Next-Generation Sequencing for Differential Diagnosis of Rapidly Progressive Diffuse Parenchymal Lung Diseases. *Frontiers in Cellular and Infection Microbiology*.

[3] Marrie T. J. (1993). Mycoplasma Pneumoniae Pneumonia Requiring Hospitalization, With Emphasis on Infection in the Elderly. *Archives of Internal Medicine*.

[4] Arab T., Malekzadegan M. R., Morante J., Cervellione K. L., Fein A. M. (2019). Nonresolving Pneumonia in the Setting of Malignancy. *Current Opinion in Pulmonary Medicine*.

[5] Fernández-Ruiz M., Castón J. J., Del Pozo J. L. (2024). How Can We Optimize the Diagnostic and Therapeutic Approach to Pneumonia? Expert Opinion-Based Recommendations. *Enfermedades Infecciosas y Microbiología Clínica*.

[6] Fernández-Ruiz M., Castón J. J., Del Pozo J. L., Aguado J. M., En Representación de Los Miembros Del Grupo Openin (2025). Optimización De Procesos Cpedytdi, How Can We Optimize the Diagnostic and Therapeutic Approach of Pneumonia? Expert Opinion-Based Recommendations. *Enfermedades Infecciosas y Microbiología Clínica*.

[7] Wei L., Luo J., Wu W. (2024). Clinical Diagnostic Value of Metagenomic Next-Generation Sequencing in Patients With Acute Infection in Emergency Department. *Heliyon*.

[8] Yao A., Wang J., Xu Q. (2024). Higher Diagnostic Value of Metagenomic Next-Generation Sequencing in Acute Infection Than Chronic Infection: A Multicenter Retrospective Study. *Frontiers in Microbiology*.

[9] Hurter T., Hanrath P. (1992). Endobronchial Sonography: Feasibility and Preliminary Results. *Thorax*.

[10] Kurimoto N., Murayama M., Yoshioka S., Nishisaka T., Inai K., Dohi K. (1999). Assessment of Usefulness of Endobronchial Ultrasonography in Determination of Depth of Tracheobronchial Tumor Invasion. *Chest*.

[11] Guan S., Xu X., Zhu X., Ge Y., Xie J., Zhou J. (2024). Diagnostic Value of rEBUS-TBLB Combined Distance Measurement Method Based on Ultrasound Images in Bronchoscopy for Peripheral Lung Lesions. *SLAS Technology*.

[12] Wang G., Zhang L., Wu H. (2017). Endobronchial Ultrasonography Using a Guide Sheath Technique for Diagnosis of Peripheral Pulmonary Lesions. *Endosc Ultrasound*.

[13] Li G., Huang J., Li Y., Feng J. (2020). The Value of Combined Radial Endobronchial Ultrasound-Guided Transbronchial Lung Biopsy and Metagenomic Next-Generation Sequencing for Peripheral Pulmonary Infectious Lesions. *Canadian Respiratory Journal*.

[14] Chen H., Bai X., Gao Y., Liu W., Yao X., Wang J. (2021). Profile of Bacteria With ARGs Among Real-World Samples From ICU Admission Patients With Pulmonary Infection Revealed by Metagenomic NGS. *Infection and Drug Resistance*.

[15] Zou X., Xu H., Hu Q. (2024). Diagnostic Efficacy of Endobronchial Ultrasound-Guided Transbronchoscopic Lung Biopsy for Identifying Tuberculous Nodules. *BMC Infectious Diseases*.

[16] Xie F., Yang H., Huang R. (2021). Chinese Expert Consensus on Technical Specifications of Electromagnetic Navigation Bronchoscopy in Diagnosing Peripheral Pulmonary Lesions. *Journal of Thoracic Disease*.

[17] Cao J., Zhou R., He Q., Zhang M., Feng C. (2024). Value of Rapid On-Site Evaluation Combined With Interventional Pulmonology Techniques in the Diagnosis of Pulmonary Cryptococcosis. *The Clinical Respiratory Journal*.

